# Physiological and Transcriptome Analysis Provide Insights into the Effects of Low and High Selenium on Methionine and Starch Metabolism in Rice Seedlings

**DOI:** 10.3390/ijms26041596

**Published:** 2025-02-13

**Authors:** Yang Yang, Jiarui Zhang, Lijuan Sun, Qin Qin, Shiyan Yang, Jun Wang, Yafei Sun, Yong Xue

**Affiliations:** 1 College of Fisheries and Life Science, Shanghai Ocean University, Shanghai 201306, China; rickyyoung2000@163.com (Y.Y.); 18363019947@163.com (J.Z.); 2 ECO—Environment Protection Research Institute, Shanghai Academy of Agricultural Sciences, Shanghai 201403, China; sunliuliu2012@126.com (L.S.); qinqin19870987@126.com (Q.Q.); 20220703@saas.sh.cn (S.Y.); junwang2018@163.com (J.W.)

**Keywords:** selenium, rice, transcriptome, physiological

## Abstract

Selenium (Se) is an essential micronutrient for the human body and is closely linked to health. Rice (*Oryza sativa* L.), as a major staple food globally, is the primary source of Se intake for humans. To better achieve Se biofortification in rice, it is crucial to understand the molecular mechanisms behind rice’s response to different Se concentrations. This study investigates the morphological and transcriptomic responses of rice seedlings to low (1 µM, LSe) and high (10 µM, HSe) Se concentrations compared to a control (CK). Morphological analyses revealed that LSe promoted growth, enhancing shoot and root length and biomass, whereas HSe treatment inhibited these parameters, indicating Se’s dual role in rice growth. Notably, the most significant promotion of rice growth was observed at the Se concentration of 1 µM. The organic Se content and antioxidant enzyme activities (SOD, POD and CAT) in rice seedlings also reached their maximum values simultaneously. Total RNA was extracted for transcriptome sequencing, and differential gene expression analysis was conducted using DESeq2. Transcriptomic sequencing highlighted distinct responses under LSe and HSe conditions. Gene ontology (GO) enrichment analysis revealed significant involvement in processes related to oxidoreductase activity and cellular structures. KEGG pathway analysis emphasized that Se treatments notably enhanced the glutathione metabolism pathway, which is crucial for antioxidant defense. Additionally, significant changes were observed in starch and sucrose metabolism and cysteine (Cys) and methionine (Met) metabolism pathways, showing upregulation under LSe treatment and downregulation under HSe. Six key genes were validated using qRT-PCR, confirming their differential expression under varied Se treatments. The Cys, Met and starch content assays as well as qRT-PCR data demonstrated that LSe promoted the synthesis and accumulation of Cys, Met and starch, supporting enhanced growth and antioxidant capacity. Conversely, HSe inhibited the synthesis and accumulation of Cys, Met and starch in rice seedlings, and these data were also consistent with the physiological phenotype. These findings provide insights into the molecular mechanisms by which rice seedlings adapt to varying Se levels, with implications for Se biofortification and stress management strategies in crops.

## 1. Introduction

Selenium (Se), an essential trace element for human and animal health, plays critical structural and enzymatic roles in various physiological functions, encompassing antioxidant defense, anti-cancer activity, anti-inflammatory responses and immune regulation [1]. Se deficiency disrupts the normal functioning of selenoproteins and Se-dependent enzymes, resulting in a cascade of complications, including reproductive dysfunction, infertility, and mental retardation [2]. Dietary Se supplementation is recognized as a safe and efficient strategy to address Se deficiency [3]. Rice (*Oryza sativa* L.), a cornerstone of global agriculture, serves as a primary source of energy and micronutrients for over half of the world’s population [4,5]. Since the late 1980s, several Asian countries have actively implemented Se fertilizer applications to cultivate Se-enriched rice, aiming to mitigate Se deficiency and enhance nutritional security [6]. Consequently, investigating the adaptive mechanisms and growth responses of rice under varying Se conditions holds profound implications for advancing food security and promoting human health.

Se is often described as a “double-edged sword” due to its dualistic biological behavior, exhibiting both essential and toxic properties depending on its concentration [7]. The threshold between Se deficiency and toxicity in living organisms is remarkably narrow, posing significant challenges for its management in biological and environmental systems [8]. At optimal concentrations, Se performs multiple pivotal roles in promoting plant growth, underscoring its essentiality in plant physiological process. Se enhances the activity and content of glutathione peroxidase (GSH-Px), a crucial enzyme in neutralizing reactive oxygen species (ROS) and lipid peroxides (LPO). This augmentation strengthens plants’ antioxidant defenses, bolstering their resilience to environmental stressors and delaying senescence [9]. Se enhances plant resilience against diverse environmental stresses by scavenging excessive free radicals generated under extreme conditions, such as high radiation, extreme temperatures, drought, and high salinity, thereby fortifying plant tolerance to adverse environments [10,11]. At excessive concentrations, Se transitions from an antioxidant to a pro-oxidant, causing an accumulation of ROS and exacerbating oxidative stress, ultimately disrupting photosynthesis in plants [12]. Excess Se disrupts photosystem reaction centers, significantly reducing photosynthetic efficiency and impairing energy conversion processes in plants [13]. High selenate concentrations induce severe phytotoxicity, markedly decreasing the fresh biomass of crops such as broccoli and cabbage [14,15]. Additionally, high Se levels significantly diminish the accumulation of flavonoids and total phenols in mustard, thereby impairing the plant’s metabolic functions [16]. Rao demonstrated that Se-binding protein 1 (SBP1) and sulfur deficiency-induced protein 2 (SDI2) are upregulated under high selenate concentrations, enhancing the tolerance of *Cardamine violifolia* to Se-induced toxicity [17]. Previous studies revealed that high Se concentrations significantly inhibit rice growth, particularly in roots, stems, and leaves, where dry biomass accumulation is notably reduced. This growth inhibition primarily arises from impaired photosynthesis and disrupted water metabolism, causing physiological drought-like symptoms in plants [18]. Excess Se significantly depletes chlorophyll, carotenoids, and protein content, visibly manifesting as chlorotic and necrotic leaf symptoms. Although antioxidant enzymes such as superoxide dismutase (SOD), perpxidase (POD) and catalase (CAT) are upregulated in response to Se toxicity, their activity is insufficient to fully mitigate the detrimental effects [19]. These findings lay a foundation for elucidating the physiological and biochemical responses of plants to high Se concentrations. However, the molecular mechanisms driving rice responses to varying Se concentrations remain inadequately understood. Consequently, further investigations into gene expression and metabolic shifts in rice are essential to unravel the molecular pathways governing rice adaptation to varying Se concentrations.

Se treatment regulates carbohydrate metabolism in rice kernels by influencing the expression of enzymes involved in sucrose and starch biosynthesis. This regulation leads to enhanced accumulation of starch and sucrose, ultimately promoting early maturity and increasing the yield of rice kernels [20]. Beyond serving as primary energy sources, starch and sucrose metabolism also play critical roles in orchestrating plant growth and developmental processes. For instance, during the early growth stages, sucrose translocation and catabolism provide essential substrates that support the development of roots and leaves, ensuring the establishment of a robust plant architecture. Moreover, these metabolic pathways interact synergistically with hormonal signals, such as abscisic acid and auxins, to modulate growth rates and shape the morphological structure of rice [21]. Under stress conditions, adjustments in starch and sucrose metabolism help sustain cellular metabolic demands and improve the resilience of rice, thereby enabling plants to better adapt to adverse environmental challenges [22].

Throughout the developmental stages of rice endosperm, amino acid metabolism—particularly Cys and Met pathways—not only facilitates seed growth but also synergistically interacts with starch and sucrose metabolism to form a nutrient-rich endosperm. This coordination is particularly prominent in polyploid rice seeds, which exhibit higher nutrient density compared to their diploid counterparts [23]. Se treatment has been shown to promote the biosynthesis of selenocysteine (SeCys) and selenomethionine (SeMet) in rice, thereby boosting the plant’s antioxidant capacity and nutritional value [24]. Cys and Met serve as precursor amino acids for the synthesis of SeCys and SeMet, underpinning key metabolic pathways in rice. To mitigate the toxicity associated with Se amino acid substitutions in proteins, SeCys undergoes methylation to form methylselenocysteine (MeSeCys), which is further metabolized into the volatile compound dimethyl diselenide. Another fraction of SeCys is enzymatically converted into Se-homocysteine via cystathionine β-lyase and transported to the chloroplast, where Met synthase catalyzes its transformation into SeMet [25,26]. During this process, SeMet and MeSeCys partially substitute Cys and Met within the sulfur metabolism pathway, highlighting their functional integration into essential biochemical networks. Cys acts as a precursor for glutathione synthesis, a pivotal antioxidant that enables rice to mitigate oxidative stress and maintain cellular homeostasis [27]. S-adenosylmethionine (SAM), a metabolite derived from Met, exhibits potent antioxidant properties and serves as a key regulatory signaling molecule, bolstering rice resilience against abiotic stresses, including salinity and drought [28]. Met participates in methylation processes mediated by SAM, encompassing the methylation of DNA, RNA, and proteins. These epigenetic modifications are crucial for regulating gene expression, thereby influencing rice growth, development, differentiation, and environmental adaptation. Methylation additionally modulates chloroplast functionality, thereby optimizing photosynthetic efficiency and driving biomass accumulation [29]. Consequently, investigating the effects of varying Se treatments on starch and sucrose metabolism, as well as Cys and Met pathways, is vital for elucidating the molecular mechanisms governing rice growth and development under diverse Se conditions.

This study aims to conduct a comprehensive investigation of differential gene expression and pivotal metabolic pathways in rice seedlings exposed to low and high Se treatments. By centering on starch and sucrose metabolism, alongside Cys and Met metabolic pathways, this research aims to elucidate the molecular mechanisms underpinning rice growth and metabolic adaptations to varying Se concentrations. These findings aim to provide critical insights into Se’s role in modulating plant health, enhancing stress resilience, and informing potential biofortification strategies to improve crop nutritional quality.

## 2. Results

### 2.1. The Effects of Low and High Se on the Phenotype of Rice Seedlings

**Figure 1:**
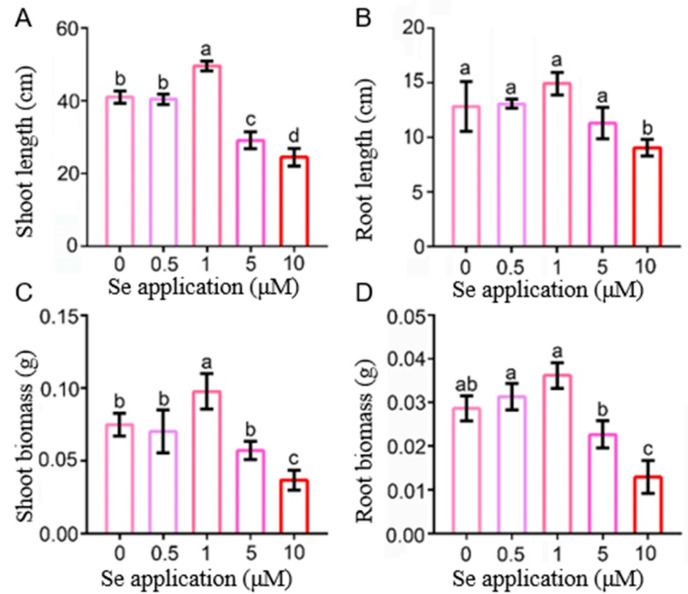
Effects of different Se concentration on the growth parameters of rice seedlings. (**A**) Shoot length, (**B**) root length, (**C**) shoot biomass, and (**D**) root biomass of rice seedlings under different Se treatments. Seedlings (14 d old) of the rice were grown hydroponically in nutrient rich solution and then under 0, 0.5, 1, 5 and 10 μM Se treatments for 14 d, respectively. Data are represented as the mean ± standard deviation (SD). Different letters indicate significant differences under different Se treatments (*p* < 0.05, Student’s *t*-test).

The level of Se concentration is crucial for the growth and development of rice seedlings. It was found that low Se had a significant growth promotion effect on rice seedlings [30]. Our findings reveal that a low Se concentration of 1 µM is optimal for promoting rice seedling growth, as evidenced by significant increases in shoot and root length and biomass, which increased by an average of 20.9%, 16.4%, 30.4%, and 28.5%, respectively, compared with 0 μM. However, at HSe concentration (10 μM), all the phenotypes of rice seedlings were significantly lower than 0 μM, resulting in an average decrease of 50.8%, 54.9%, 40.2% and 29.4% (Figure 1). This suggests that low Se concentrations facilitate the growth and development of rice seedlings, while excessive Se may have toxic effects on plants and inhibit the growth and development of rice seedlings.

### 2.2. The Effects of Low and High Se on the Concentration of Se in Rice Seedings

**Figure 2:**
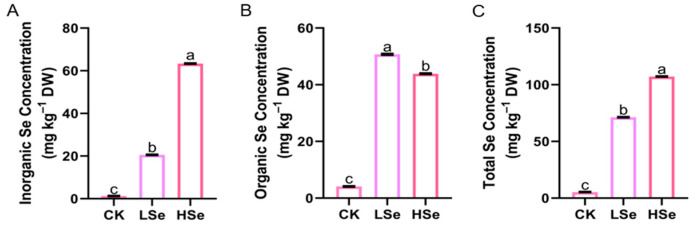
Se content of different species in rice seedlings under different Se treatments. (**A**) Total Se concentration, (**B**) inorganic Se concentration, and (**C**) organic Se concentration of rice seedlings under different Se treatments. Seedlings (14 d old) of the rice were grown hydroponically in nutrient rich solution and then under 0, 1, and 10 μM Se treatments for 14 d, respectively. Data are represented as the mean ± standard deviation (SD). Different letters indicate significant differences under different Se treatments (*p* < 0.05, Student’s *t*-test).

To investigate the effect of different Se concentrations on the Se content in rice seedlings, the total Se content in the seedlings was quantified (Figure 2A). The results revealed that Se application significantly enhanced the total Se content in the rice seedlings. The total Se content in the seedlings under the HSe treatment peaked at 1.51 times that of the LSe treatment and 17.83 times that of the CK treatment. Additionally, the inorganic and organic Se contents in the rice seedlings were also measured. The results indicated that both LSe and HSe treatments significantly elevated the inorganic Se content in the seedlings relative to the CK, with the most pronounced increase observed under the HSe treatment (Figure 2B). Moreover, Se application significantly increased the organic Se content in the rice seedlings, with the highest content observed under the LSe treatment, which was 1.16 times greater than that observed under the HSe treatment (Figure 2C).

### 2.3. The Effects of Low and High Se on the Antioxidant Enzyme Activities of Rice Seedings

**Figure 3:**
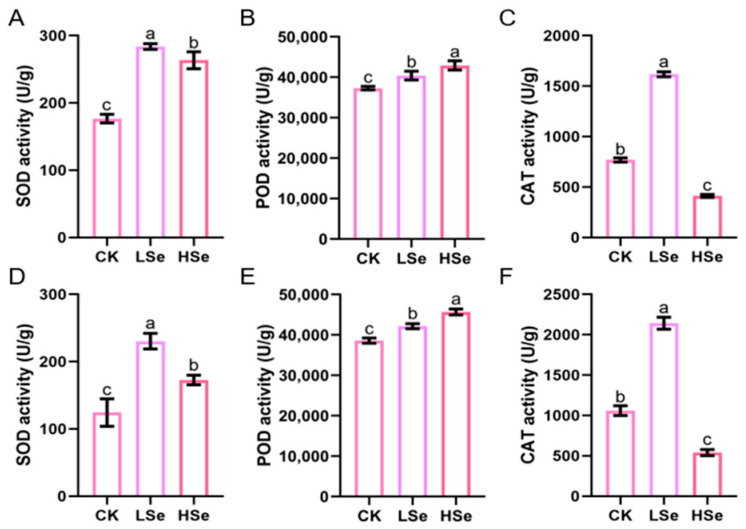
Effects of different Se concentration on the antioxidant enzyme activities of rice seedlings. (**A**,**D**) SOD activity, (**B**,**E**) POD activity, (**C**,**F**) CAT activity of rice seedlings under different Se treatments. (**A**–**C**) shows shoot data; (**D**–**F**) shows root data. Seedlings (14 d old) of the rice were grown hydroponically in nutrient rich solution and then under 0, 1, and 10 μM Se treatments for 14 d, respectively. Data are represented as the mean ± standard deviation (SD). Different letters indicate significant differences under different Se treatments (*p* < 0.05, Student’s *t*-test).

Se exhibits a dual role in plants, acting as an antioxidant at low concentrations while inducing oxidative stress at high concentrations [7]. To better understand this dose-dependent effect of Se, we evaluated the activities of key antioxidant enzymes, SOD, POD, and CAT, in rice seedlings subjected to different Se treatments (Figure 3). These enzymes serve as reliable biochemical markers for assessing the oxidative balance and stress tolerance of plants. As the Se concentration increased, the activities of SOD and CAT in both the shoots and roots of rice seedlings showed a trend of increasing followed by decreasing (Figure 3A,C,D,F). Remarkably, under the HSe treatment, the SOD activity in the shoots and roots of rice seedlings was increased by 49.4% and 38.7%, respectively, compared to the CK, whereas the CAT activity in the shoots and roots under the same treatment decreased by 46.3% and 48.9%, respectively, compared to the CK (Figure 3B,E). As the Se application increased, the POD activity in the shoots and roots of rice seedlings showed a consistent upward trend, peaking under the HSe treatment, where the average increase was 18% and 8% higher than that observed in the CK and LSe, respectively.

### 2.4. The Different Impacts of Low and High Se on the Differential Gene Expression in Rice Seedlings

The mechanism of association between different Se concentrations and rice seedlings was analyzed by transcriptome sequencing. Table S2 provides a comprehensive summary of RNA-seq data and reads mapping across different sample conditions (CK, LSe, HSe) for both root and shoot tissues. The clean reads for all samples are consistently high, with Q30 values exceeding 95%, indicating high sequencing accuracy. GC content varies between 50.64% and 53.06%, reflecting consistency across the samples. The proportion of total mapped reads ranges from 92.85% to 98.25%, while uniquely mapped reads range from 88.82% to 93.54%, demonstrating robust alignment efficiency. These metrics collectively indicate high-quality RNA-seq data, suitable for reliable downstream analyses.

Principal component analysis (PCA) was conducted based on expression data (Figure S1), with principal component 1 (PC1) and principal component 2 (PC2) explaining 68.33% and 11.17% of the total variation, respectively. The six sample groups (CK-R, CK-S, HSe-R, HSe-S, LSe-R, LSe-S) were significantly separated in the two-dimensional principal component space, indicating notable differences in their characteristic variables. This distribution reveals significant differences among the treatment groups. The reproducibility and correlation of the three replicates under different treatment concentrations were high, ensuring the reliability of subsequent analyses.

**Figure 4:**
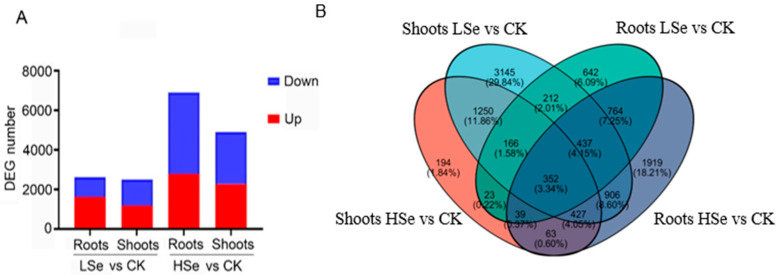
Differentially expressed genes (DEGs) in rice roots and shoots under Se treatments. (**A**) Number of upregulated and downregulated DEGs in roots and shoots of rice seedlings under LSe and HSe conditions compared to the CK. Red bars indicate upregulated genes, and blue bars indicate downregulated genes. (**B**) Venn diagram showing the overlap of DEGs among different comparisons (roots LSe vs. CK, shoots LSe vs. CK, roots HSe vs. CK, and shoots HSe vs. CK). Each section of the diagram represents the number and percentage of shared and unique DEGs in the respective conditions.

The transcriptome sequencing results revealed that there were 2514 DEGs comprising 1161 upregulated genes and 1353 DEGs downregulated genes in the shoots and 1613 upregulated genes and 1022 downregulated genes in the roots under low Se treatment (Figure 4A). There were 2784 upregulated genes and 4111 downregulated genes in the shoots and 2271 upregulated genes and 2636 downregulated genes in the roots under high Se treatment. The above results indicated that high concentrations of Se account for an increase in the number of DEGs in rice seedlings. Notably, only the number of upregulated genes was more than downregulated genes in the roots under low Se treatment, while the number of downregulated genes was more than upregulated genes in all three other groups. The Venn analysis showed that the number of genes unique to each of the four treatment groups was as follows: 194 in the shoot of HSe vs. CK group, 3145 in the shoot of LSe vs. CK group, 642 in the root of LSe vs. CK group, and 1919 in the root of Hse vs. CK group (Figure 4B). There were 352 genes shared among the four groups which illustrated the cross-impact of different Se treatments on various metabolic pathways in rice.

### 2.5. GO Enrichment Analysis of DEGs

**Figure 5:**
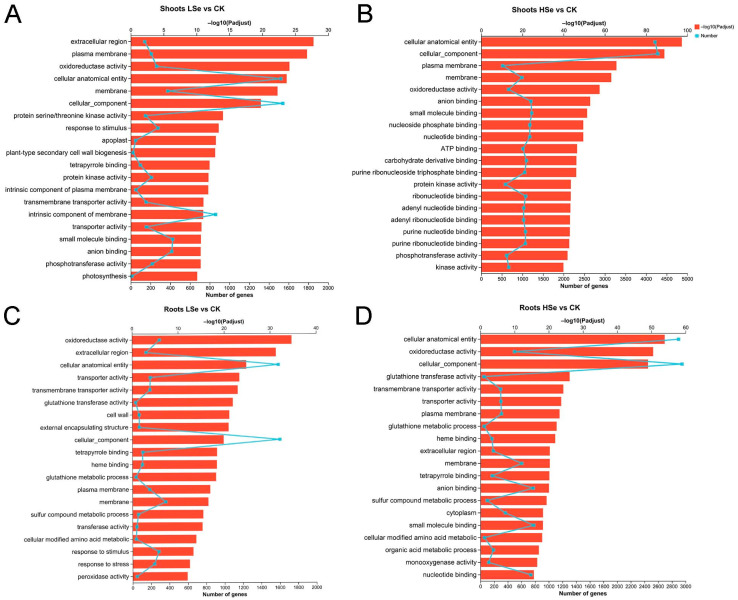
GO enrichment analysis of differentially expressed genes (DEGs) in rice shoots and roots under LSe and HSe treatments compared to CK. (**A**) Shoots LSe vs. CK, (**B**) shoots HSe vs. CK, (**C**) roots LSe vs. CK, and (**D**) roots HSe vs. CK. The x-axis shows the number of genes, and the y-axis lists enriched GO terms. The blue line represents the −log10 (Padjust) values, indicating the significance of enrichment.

Gene ontology (GO) enrichment analysis was carried out to further characterize the main biological functions of DEGs in rice seedlings under Se treatments. All the DEGs can be divided into three categories, including biological process, molecular function, and cellular component. The top 20 significantly enriched GO terms were shown in Figure 5. In the shoot of the LSe vs. CK groups, key terms included “response to stimulus” and “plant-type secondary cell wall biogenesis” under biological processes; “extracellular region”, “plasma membrane”, and “cellular anatomical entity” under cellular components, and “cellular processes”, “oxidoreductase activity”, and “protein serine/threonine kinase activity” under molecular function (Figure 5A). As shown in the shoot of the HSe vs. CK groups, the differential genes were involved in cellular component and molecular function, with molecular function accounting for the majority of them. “Cellular anatomical entity”, “cellular component”, and “plasma membrane” were the three most enriched subcategory in the cellular component. Oxidoreductase activity was the most enriched subcategory in the molecular function (Figure 5B).

Meanwhile, the DEGs are also divided into three categories in roots of rice. There were five, seven, and eight enriched subcategories belonging to the categories of biological process, molecular function and cellular component. The most enriched subcategory in biological process was glutathione metabolic process the LSe vs. CK groups, followed by “sulfur compound metabolic process” and cellular modified amino acid metabolic process. Extracellular region was the most enriched subcategory in cellular components. Among the molecular functions, the three most enriched subcategories were oxidoreductase activity, transporter activity and transmembrane transporter activity (Figure 5C). The three most enriched subcategories in the root of the HSe vs. CK groups were glutathione metabolic process, sulfur compound metabolic process and cellular modified amino acid metabolic process. Oxidoreductase activity was the most enriched subcategory in molecular function (Figure 5D).

### 2.6. Kyoto Encyclopedia of Genes and Genomes (KEGG) Enrichment Analysis of DEGs

**Figure 6:**
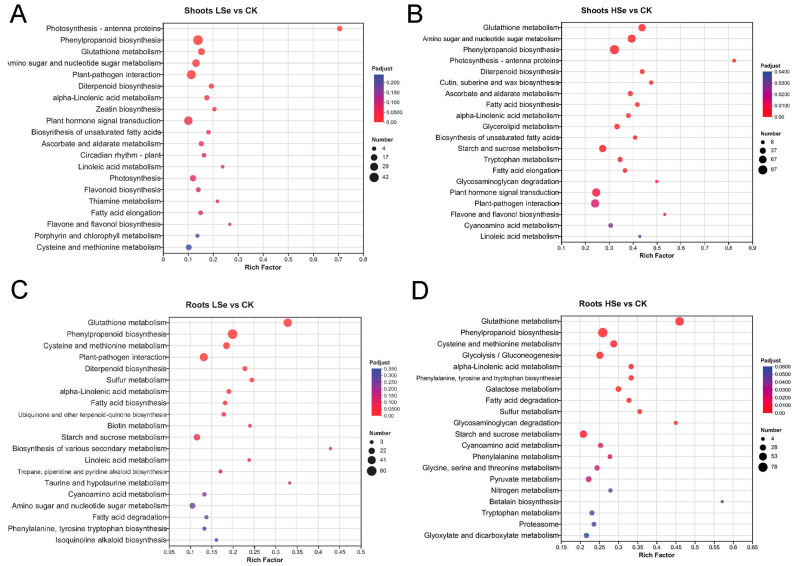
KEGG pathway enrichment analysis of differentially expressed genes (DEGs) in rice shoots and roots under different treatments. (**A**) Shoots LSe vs. CK, (**B**) shoots HSe vs. CK, (**C**) roots LSe vs. CK, and (**D**) roots HSe vs. CK. The x-axis represents the rich factor, indicating the ratio of DEGs in each pathway, while the y-axis lists the enriched KEGG pathways. Dot size reflects the number of DEGs, and the color gradient represents the significance level (Padjust), with red indicating higher significance.

In order to assign DEGs to cellular pathways, pathway enrichment analysis based on KEGG was performed. The DEGs significantly enriched pathways were calculated using hypergeometric distribution based on the whole genome. There are four KEGG pathway categories: organismal systems, metabolism, environmental information processing and genetic information processing. The top 20 significantly enriched KEGG terms were shown in Figure 6. Plant–pathogen interaction and plant hormone signal transduction were the only enriched processes in organismal systems and environmental information processing in the shoot of the LSe vs. CK groups (Figure 6A). In the category of metabolism, most of the pathways were highly overrepresented, including photosynthesis-antenna proteins, phenylpropanoid biosynthesis, glutathione metabolism, amino sugar and nucleotide sugar metabolism, diterpenoid biosynthesis and alpha-linolenic acid metabolism. No significant enrichment was found in genetic information processing. In addition, we found plant–pathogen interaction and plant hormone signal transduction were the only enriched processes in organismal systems and environmental information processing in the shoots of the HSe vs. CK groups which was the same in the shoot of the LSe vs. CK group. In the category of metabolism, glutathione metabolism was the most overrepresented, followed by amino sugar and nucleotide sugar metabolism, phenylpropanoid biosynthesis, diterpenoid biosynthesis and fatty acid biosynthesis (Figure 6B).

The most enriched category in the four groups was metabolism. “Plant–pathogen interaction” was the only enriched item in organismal systems in the root of the LSe vs. CK groups (Figure 6C). In regard to metabolism, Glutathione metabolism was the most highly overrepresented, followed by phenylpropanoid biosynthesis, Cys and Met metabolism, Diterpenoid biosynthesis, Sulfur metabolism, alpha-linolenic acid metabolism and Fatty acid biosynthesis. No enrichment was found in environmental information processing and genetic information processing. As shown in Figure 6D, the enriched items were involved in metabolism and genetic information processing, with metabolism accounting for the majority of them. “Glutathione metabolism”, “Phenylpropanoid biosynthesis”, and “Cys and Met metabolism” were the three most enriched item in the metabolism. Proteasome was the most enriched item in the genetic information processing. Interestingly, we found starch and sucrose metabolism and Cys and Met metabolism were upregulated in the root of the LSe vs. CK groups and downregulated in the root of the HSe vs. CK group. In addition, we further analyzed the DEGs in the roots to screen for genes that were upregulated under low Se treatment and downregulated under high Se treatment. The KEGG enrichment revealed that starch and sucrose metabolism and Cys and Met metabolism were the most enriched pathway (Figure S2).

**Figure 7:**
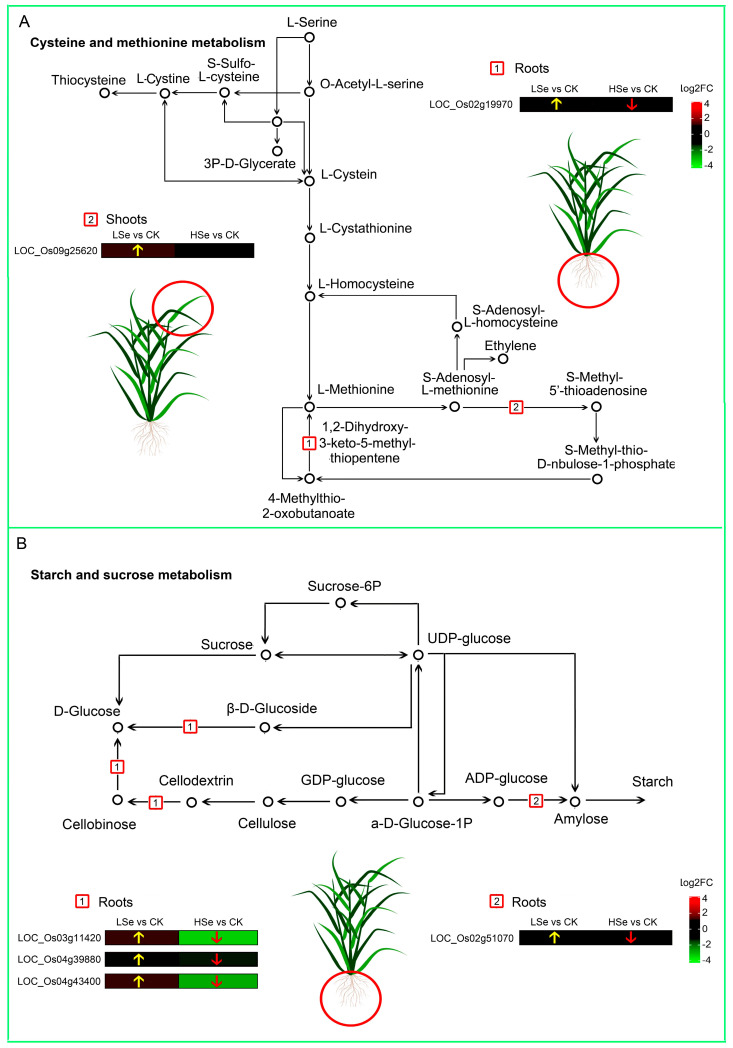
Schematic representation of Met, Cys, and starch metabolism pathways in rice under LSe and HSe treatment. The pathways show Cys and Met metabolism (**A**) and starch and sucrose metabolism (**B**), with corresponding differentially expressed genes highlighted by log_2_ fold change (log_2_FC) color-coded boxes. Seedlings (14-day-old) of rice were grown hydroponically in nutrient rich solution and then subjected to 7 d 0 (CK), 1 (LSe), and 10 (HSe) μM Se treatments. The yellow arrows highlight the upregulation of genes, while the red arrows emphasize the downregulation of genes. The red boxes and the different numbers within them represent various reaction pathways within that metabolism pathway.

In Cys and Met metabolism, two genes, LOC_Os09g25620 and LOC_Os02g19970 were all upregulated by low Se treatment and downregulated by high Se treatment (Figure 7A). In addition, LOC_Os03g11420, LOC_Os04g39880, LOC_Os04g43400 and LOC_Os02g51070 in starch and sucrose metabolism were upregulated by low Se treatment (Figure 7B), while they were all reduced by high Se treatment. These results imply these genes may play a significant role in the response of rice seedlings to different concentrations of Se treatments, and the pathways involving the expression of these genes are also crucial for the response of rice seedlings to Se stress.

### 2.7. The Effects of High and Low Se on the Accumulation of Starch, Cys and Met

**Figure 8:**
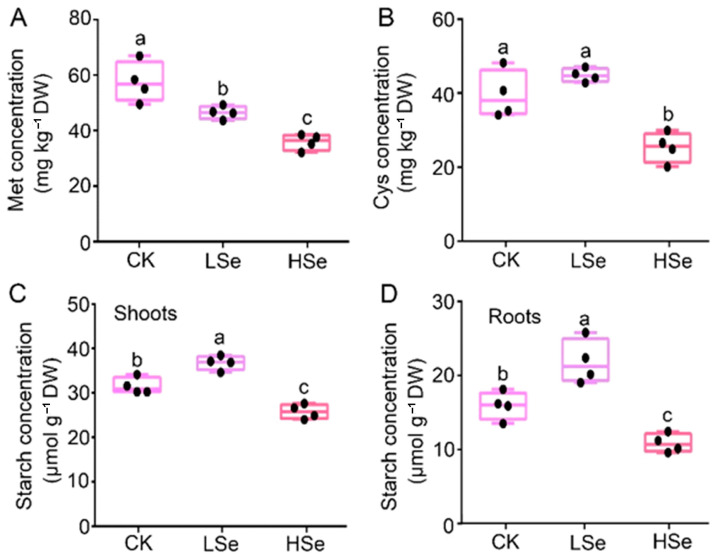
Concentrations of Met, Cys, and starch in rice seedlings under CK, LSe and HSe treatments. (**A**) Met concentration, (**B**) Cys concentration, (**C**) starch concentration in shoots, and (**D**) starch concentration in roots. Data are represented as the mean ± standard deviation (SD). Different letters indicate significant differences under different Se treatments (*p* < 0.05, Student’s *t*-test).

The Met, Cys and starch contents in rice seedlings under different Se treatments were determined. The results indicate that Met concentration was highest under CK treatment, followed by LSe treatment, and was lowest under HSe treatment, showing a significant decreasing trend (Figure 8A). Cys concentration showed no significant difference between CK and LSe treatments, both of which were higher than the HSe treatment, which exhibited a significantly reduced concentration (Figure 8B). The distribution of starch concentration in both the shoots and roots of seedlings demonstrated that LSe treatment reached the highest levels, followed by CK treatment, while HSe treatment resulted in the lowest levels, showing a significant decrease (Figure 8C,D). These findings suggest that LSe significantly promoted the accumulation of starch, Cys, and Met in rice seedlings, whereas HSe inhibited the accumulation of these substances, highlighting the significant impact of Se concentration on the metabolism of rice seedlings.

### 2.8. Validation of RNA Sequencing Date by Quantative Real-Time PCR

**Figure 9:**
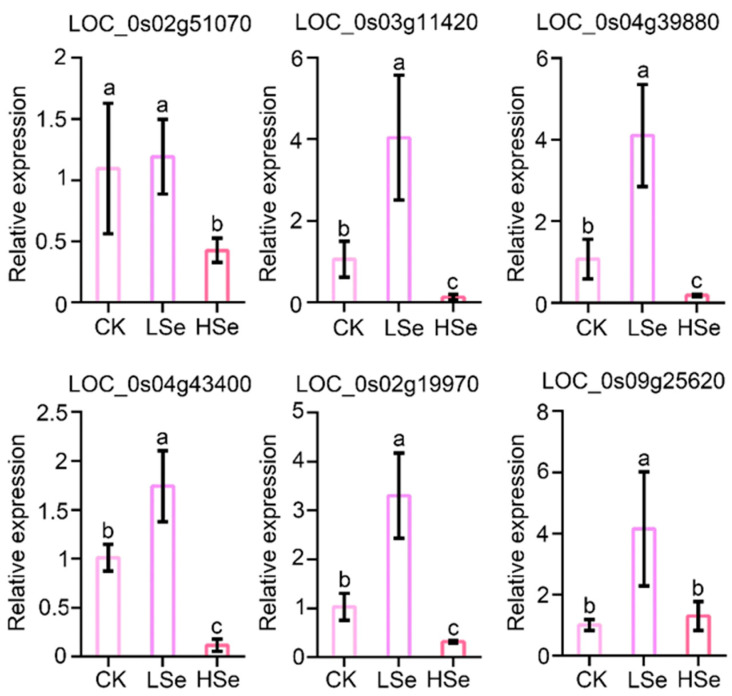
Relative expression levels of genes related to Cys/Met metabolism and starch/sucrose metabolism. Gene expression is shown as the mean ± standard deviation (SD). *OsACTIN* was used as an internal control. The relative expression of LOC_Os02g51070, LOC_Os03g11420, LOC_Os04g39880, LOC_Os04g43400, LOC_Os02g19970 and LOC_Os09g25620 under CK conditions was standardized to 1. Different letters indicate significant differences under different Se treatments (*p* < 0.05, Student’s *t*-test).

A total of six genes were randomly selected and validated by quantitative real-time (qRT-PCR) to confirm the reliability of sequencing date (Figure 9). Among them, one gene encodes conserved peptide uORF-containing transcript (CPuORF8) (LOC_Os09g25620), one gene encodes aminotransferase, one gene encodes starch synthase (LOC_Os02g51070) and each of the three genes encodes *Oryza sativa* β-glucosidase6 (Os3bglu6), *Oryza sativa* β-glucosidase 12 (Os4bglu12) and *Oryza sativa* β-glucosidase 17 (Os4bglu17), members of the GH1 family (LOC_Os03g11420, LOC_Os04g39880, LOC_Os04g43400). The genes LOC_0s02g51070, LOC_0s03g11420, LOC_0s04g39880, LOC_0s04g43400, LOC_0s02g19970, and LOC_0s09g25620 exhibit significantly higher expression levels in the LSe treatment group compared to the CK and HSe groups. Overall, the trend indicated that these genes were upregulated under low Se conditions and significantly suppressed under HSe conditions. The results showed that the changes were basically consistent with the results of transcriptome sequencing. These selected and validated genes provide a more comprehensive understanding of the gene response patterns under Se stress and their contributions to the overall metabolic network.

## 3. Discussion

Se plays an important physiological role in plant growth. Although Se is not an essential element for plants, an appropriate amount of Se can enhance the plant’s antioxidant capacity, improve stress resistance, promote growth, and enhance the nutritional quality of crops [31]. Se can enhance photosynthesis in rice, allowing the plant to accumulate more photosynthetic products, thus facilitating rapid shoot growth [32]. The results of this study indicate that low Se concentrations promote rice seedling growth, while high Se concentrations inhibit both biomass and root growth (Figure 1). This finding is consistent with previous studies, showing that 0.5–1 mg/kg Se promotes significant growth in rice seedlings [33]. Additionally, the oxidative damage induced by HSe concentrations can affect energy metabolism and reduce growth rates [34]. Our results further confirm the benefits of low Se on rice seedling growth, as well as the toxic effects of high Se, particularly 1 µM Se, which showed the most pronounced promotion of growth.

In this study, the effects of different Se concentrations on the Se content in rice seedlings were investigated. The results demonstrated that Se application significantly enhanced the total Se content in seedlings, with the highest accumulation observed under the HSe treatment (Figure 2A). This aligns with previous studies indicating that higher Se concentrations lead to greater Se uptake in rice [35]. Moreover, the low Se treatment promoted the synthesis of organic Se, as evidenced by a significantly higher organic Se content compared to high Se treatment (Figure 2C). This suggests that lower Se concentrations may favor the synthesis of organic Se species, such as SeMet and SeCys, which are more beneficial for human nutrition [36].

The antioxidant enzyme system plays a crucial role in alleviating oxidative stress in plants. Enzymes such as SOD, CAT, and POD can scavenge ROS and protect cells from damage caused by oxidative stress [37]. It was found that the activities of SOD and CAT in both shoots and roots displayed a trend of increasing followed by decreasing as Se concentration increased (Figure 3A,C,D,F). Under HSe treatment, SOD activity in shoots and roots increased by 49.4% and 38.7%, respectively, compared to CK, indicating an enhanced scavenging of superoxide radicals. However, CAT activity decreased by 46.3% and 48.9%, respectively, under the same treatment, suggesting that excessive Se may overwhelm the enzyme system, leading to its inactivation or degradation. POD activity, in contrast, exhibited a consistent upward trend with increasing Se levels, peaking under HSe treatment (Figure 3B,E). This aligns with studies showing that POD activity often increases under stress conditions to compensate for the loss of other antioxidant enzymes [38].

Transcriptome analysis revealed significant differences in DEGs in different tissues of rice under high and low Se treatments (Figure 4). Low Se treatment promoted rice seedling growth, leading to more upregulated genes than downregulated ones, thus altering the biological processes. GO enrichment analysis is instrumental in highlighting key biological processes involved in response to stress conditions. For instance, in *Pueraria lobata*, transcriptome analysis showed that Se stimulated the upregulation of genes involved in signal transduction and stress response, such as phosphate transporter and sulfate transporter genes, which regulate Se metabolism and accumulation [39]. In celery, Se treatment enriched secondary metabolic pathways, especially genes related to phenylpropanoid biosynthesis, indicating that Se may enhance plant resistance through secondary metabolic pathways [40]. Our research found that GO enrichment analysis showed significant enrichment of terms like “cellular anatomical entity” and “oxidoreductase activity” in both roots and shoots under low Se treatment, while high Se treatment enriched pathways related to “glutathione metabolism” in roots (Figure 5). Notably, the enrichment of glutathione metabolism in roots suggests that roots are the primary organs for Se uptake and conversion in plants and that they may require additional antioxidant mechanisms to mitigate Se stress and maintain cellular redox homeostasis when exposed to exogenous Se. Previous studies have also shown similar trends, confirming that appropriate Se may generate ROS during root metabolism, which can activate the glutathione metabolic pathway in roots [41].

KEGG pathway analysis further revealed that Se treatment significantly upregulated pathways related to “glutathione metabolism”, “phenylpropanoid biosynthesis”, and “alpha-linolenic acid metabolism” in both shoots and roots (Figure 6). These pathways play key roles in the antioxidant response and stress tolerance under Se treatment [3,42,43]. Additionally, genes related to starch and sucrose metabolism were significantly upregulated under low Se treatment (Figure S2). Starch and sucrose metabolism play a crucial role in plant growth. Under drought and salt stress conditions, plants regulate sucrose metabolism to increase the accumulation of storage substances, thereby enhancing stress resistance [44]. Previous study also showed that Se treatment promotes the accumulation of starch and soluble sugars in potatoes, indicating a positive effect of Se on carbon metabolism and yield [45]. Se treatment aids potato growth under heavy metal stress by regulating carbohydrate metabolism, including starch and sucrose metabolism [46]. This indicates that the upregulation of genes related to starch and sucrose metabolism under low Se treatment may lead to increased carbohydrate synthesis and accumulation. Plants may support growth and development by enhancing the storage of carbohydrates. This view was corroborated by the determination of starch content in rice (Figure 8C,D). While high Se levels may result in oxidative stress or metabolic disorders, inhibiting starch and sucrose synthesis. This could be a protective mechanism by which plants reduce energy consumption or limit the accumulation of harmful metabolic by products.

Meanwhile, Cys and Met metabolism showed a trend similar to starch and sucrose metabolism. Cys contributes to the synthesis of glutathione (GSH), helping plants cope with oxidative stress and protecting cells from damage by free radicals. Additionally, Cys participates in processes such as photosynthesis and sulfur metabolism, thereby regulating plant growth, development, and stress resistance [47]. Met, beyond being a fundamental component of proteins, is involved in various critical methylation reactions through S-adenosylmethionine (AdoMet), regulating DNA methylation and plant hormones like ethylene [48]. In this study, we found that genes related to Cys and Met metabolism were upregulated in the root of the LSe vs. CK groups and downregulated in the root of the HSe vs. CK groups (Figure 7A), supported by measurements of Cys and Met content (Figure 8A,B). This indicates that low Se enhances tolerance to ROS and supports stress adaptation, consistent with changes in antioxidant enzyme activities (Figure 3). Conversely, the oxidative stress caused by high Se may suppress Cys and Met metabolic pathways, likely as a protective mechanism to prevent overaccumulation of toxic Se derivatives. While the reduced Met content may stem from Se-induced stress and metabolic disorders. These findings provide new insights into the adaptive metabolic strategies under Se stress and highlight the Cys and Met metabolism pathway as a potential target for enhancing Se tolerance in crops.

Starch synthase is primarily responsible for synthesizing different types of starch in rice to ensure energy storage during grain development. Studies have shown that in vegetative organs such as rice leaves, enzymes like soluble starch synthase IIIb (SSIIIb) are involved in the synthesis of temporary starch, which can be converted into energy under stress or high growth demand [49]. Under drought stress, the expression and activity of AGPase and sucrose synthase genes in rice grains increase to promote the accumulation of storage starch, ensuring grain development under water-deficient conditions [50]. Our study found that genes encoding starch synthases were upregulated under low Se treatment and downregulated under high Se treatment. This indicates that changes in the expression of starch synthase-encoding genes help modulate starch synthesis under different Se treatments, optimizing energy storage and utilization in rice.

GH1 β-glucosidase is involved in hormone regulation, stress response, and ell wall remodeling in rice [51,52]. GH1 enzymes promote tissue development and help maintain plant structure. In Arabidopsis, GH1 enzymes are upregulated under high salinity, aiding in salt stress tolerance [53]. Our study found that Os3BGlu6, Os4BGlu12 and Os4BGlu17, which are members of the GH1 family, were predominantly upregulated in rice shoots under low Se treatment and downregulated under high Se treatment. Ref. [54] showed that Os3BGlu6 enhances drought resistance and photosynthetic efficiency by hydrolyzing ABA-glucose ester. Impaired expression of Os3BGlu6 leads to decreased ABA levels, reducing drought resistance and photosynthetic efficiency. Additionally, Os4BGlu12 is highly expressed during germination and maturation, playing a key role in these stages [55]. The significant differential expression of GH1 genes in rice roots under varying Se treatments may underscore their critical role in plant defense and adaptation to stress.

Aminotransferases play a crucial role in amino acid metabolism, osmotic regulation, and antioxidant functions, contributing to enhanced stress tolerance and growth stability in plants [56]. Se treatment promotes metabolic product synthesis, stress resistance, and protein content in grains [57]. The study of naturally Se-enriched rice and non-Se-enriched rice demonstrated that Se-enriched rice exhibited higher antioxidant activity and amino acid metabolism levels, highlighting the role of aminotransferases in amino acid metabolism and stress resilience [58]. Our research found that aminotransferase genes were upregulated under low Se treatment and downregulated under high Se treatment. This suggests that lower Se concentrations enhance rice growth and metabolism by stimulating aminotransferase activity. Conversely, higher Se concentrations may impair protein synthesis pathways, resulting in the downregulation of aminotransferases and associated metabolic pathways, thus slowing down growth and adaptability. This observation is consistent with previous studies indicating that Se treatment enhances the synthesis and metabolism of essential amino acids in rice, likely by stimulating aminotransferase activity. This metabolic modulation supports cellular growth and improves stress tolerance [59].

Conserved Peptide Upstream Open Reading Frames (CPuORFs) are a special class of upstream open reading frames (uORFs) whose encoded peptides are conserved across different species and are primarily involved in regulating gene translation [60]. Studies have shown that CPuORFs can play a regulatory role in gene expression through specific conserved peptide sequences, often in response to small molecule signals and environmental stress [61]. Our research identified that CPuORF8, a member of the CPuORF gene family, may play an important role in rice in response to Se treatment as an environmental signal. Prior to this study, no research had reported the regulation of these genes in rice under Se treatment. Our findings provide a reference for future investigations.

## 4. Materials and Methods

### 4.1. Rice Cultivation and Treatment Methods

The experiment was conducted in April–June 2024 in the greenhouse of the ECO—Environment Protection Research Institute, Shanghai Academy of Agricultural Sciences, Shanghai, China. The rice seeds (*Oryza sativa* L., Nipponbare) were soaked at 30 °C for 48 h, washed with aseptic water for three times, germinated at 35 °C in the dark for 24 h, and transferred into a cultivation box to raise seedlings. The Seedlings were then transplanted and cultured in nutrient solution containing different concentrations of Se. The nutrient solution was composed of (µmol·L^−1^): Ca(NO_3_)_2_·4H_2_O 183, KNO_3_ 91, KH_2_PO_4_ 100, (NH_4_)_2_SO_4_ 183, MgCl_2_·6H_2_O 274, MnSO_4_·H_2_O 1, (NH_4_)_6_Mo_7_O_24_·4H_2_O 1, ZnSO_4_·7H_2_O 1, CuSO_4_·5H_2_O 0.2, H_3_BO_3_ 3, Fe(III)-EDTA 60. The Se treatments included a control (0 µM Se) and increasing concentrations (1 and 10 µM Se) as sodium selenite. Plants were grown in a greenhouse with a 16/8-h light/dark cycle at 28 °C/22 °C and 60–70% relative humidity. Change the nutrient solution treated by hydroponics every 3 days, and pH is adjusted to 5.5 (±0.1). After 14 days of treatment, the samples were taken and the indexes were determined and analyzed. Shoot and root were harvested and frozen with liquid nitrogen, respectively, and then stored in the refrigerator at −80 °C for gene expression analysis.

### 4.2. Detection of Total Se and Different Forms of Se in Rice Seedlings

Total Se content: the samples were placed in a 105 °C oven for 20 min to deactivate enzymes and then dried at 90 °C to a constant weight. Afterward, the samples were ground and passed through a 0.15 mm sieve. The total Se content was determined using the hydride generation-atomic fluorescence spectrometry (HG-AFS) method.

Organic Se content: Following the method described by [62], 3 g of the sample was weighed and mixed with 3.5 mL of 4 mol/L hydrochloric acid solution. The mixture was heated in a boiling water bath at 100 °C for 10 min, followed by centrifugation at 4 °C at a speed of 2500 r/min for 10 min. The supernatant was diluted to 10 mL with ultrapure water, and the inorganic selenium content was determined using HG-AFS. The organic Se content was calculated as the difference between the total Se content and the inorganic Se content.

### 4.3. Determination of Antioxidant Enzyme Activities

Rice seedling samples were collected under 2 weeks different Se concentration treatments for transcriptome sequencing analysis. The rice seeding samples were divided into shoot and root, frozen quickly with liquid nitrogen and stored at −80 °C. The activities of enzymes including SOD, POD and CAT were determined through biochemical analysis kits (Sangon Biotech, Shanghai, China) according to the manufacturer’s instructions.

### 4.4. Transcriptome Sequencing Analysis

#### 4.4.1. Sampling and RNA Extraction

Rice seedling samples were collected under 2 weeks different Se concentration treatments for transcriptome sequencing analysis. The rice seeding samples were divided into shoot and root, frozen quickly with liquid nitrogen and stored at −80 °C for RNA extraction.

Total RNA was extracted from the tissue using TRIzol^®^ Reagent according the manufacturer’s instructions. Then, RNA quality was determined by 5300 Bioanalyser (Agilent Technologies, Santa Clara, CA, USA) and quantified using the ND-2000 (NanoDrop Technologies, Wilmington, DE, USA). All the RNAs were processed using the following parameters: concentration > 10 ng, OD260/280 = 1.8~2.2, 28S:18S ≥ 1.0 and RIN > 6.5 or concentration > 1 μg, OD260/280 = 1.8~2.2, OD260/230 ≥ 2.0, RQN ≥ 6.5, 28S:18S ≥ 1.0.

#### 4.4.2. cDNA Library Construction and RNA Sequencing

RNA purification, reverse transcription, library construction and sequencing were performed at Shanghai Majorbio Bio-pharm Biotechnology Co., Ltd. (Shanghai, China) according to the manufacturer’s instructions. The rice RNA-seq transcriptome library was prepared following Illumina^®^ Stranded mRNA Prep, Ligation (San Diego, CA, USA) using 1 μg of total RNA. Briefly, messenger RNA was isolated according to polyA selection method by oligo(dT) beads and then fragmented by fragmentation buffer firstly. Secondly, double-stranded cDNA was synthesized with random hexamer primers. Then, the synthesized cDNA was subjected to end-repair, phosphorylation and adapter addition according to library construction protocol. Libraries were size selected for cDNA target fragments of 300–400 bp use magnetic beads followed by PCR amplified for 10–15 PCR cycles. After quantified by Qubit 4.0, the sequencing library was performed on NovaSeq X Plus platform (PE150) using NovaSeq Reagent Kit (Illumina, San Diego, CA, USA).

#### 4.4.3. Sequence Assembly and Gene Annotation and Quantification

The raw paired end reads were trimmed and quality controlled by fastp [63] with default parameters. Then, clean reads were separately aligned to reference genome with orientation mode using HISAT2 (version 2.2.1) [64] software. The mapped reads of each sample were assembled by StringTie (version 2.2.1) [65] in a reference-based approach.

#### 4.4.4. Differentially Expressed Genes (DEGs) and Their GO and KEGG Pathway Enrichment Analyses

To identify DEGs (differential expression genes) between two different samples, the expression level of each transcript was calculated according to the transcripts per million reads (TPM) method. RSEM [66] was used to quantify gene abundances. Essentially, differential expression analysis was performed using the DESeq2 (version 1.26.0) [67]. DEGs with |log_2_FC| ≥ 1 and FDR < 0.05 (DESeq2) were considered to be significantly different expressed genes. In addition, functional-enrichment analysis including GO and KEGG were performed to identify which DEGs were significantly enriched in GO terms and metabolic pathways at Bonferroni-corrected *p*-value < 0.05 compared with the whole-transcriptome background. GO functional enrichment and KEGG pathway analysis were carried out by Goatools and Python scipy.

### 4.5. qRT-PCR Analysis

Six DEGs involved in starch and sucrose metabolism and Cys and Met metabolism were selected for qRT-PCR and used in validating the RNA-seq results. Primers were designed with Primer Premier 5 software (Premier Biosoft International, Palo Alto, CA, USA). The primers are listed in Table S1. Detailed information for qRT-PCR was the same as that of our previous study [68]. 18s-rRNA gene was selected as the internal reference gene, and the 2^−ΔΔCt^ method [69] was used in determining the relative expression levels of target genes.

### 4.6. The Analysis of Amino Acid and Starch of Rice Seedlings

The samples were prepared as follows: 2 g of frozen rice sample was taken, 10 mL solution (70% (*v*/*v*) chloroform and 30% (*v*/*v*) methanol) was added, it was grinded into homogenate under liquid N condition, free amino acid was extracted with 8 mL of water, the water layer was extracted after 3 times, it was let to stand on ice for 30 min, then 500 µL supernatant was added and absorbed. The supernatant was filtered through a 0.45-µm micropore filter and loaded into a sample vial. Then, the automatic pre-column derivatization was used and single free amino acid concentrations were measured using Agilent 1260 High Performance Liquid Chromatography (Palo Alto, CA, USA).

The rice seedlings were freezedried and weighted. The dry seeds were extensively ground into flour. The soluble sugar was extracted from flour in 80% (*v*/*v*) ethanol for 30 min at 80 °C and then centrifuged at 5000× *g* for 10 min. The resulting pellet was further washed two times with 80% ethanol. The ethanol-insoluble residue was used for total starch content determination by using Megazyme Total Starch Assay Kit (K-TSTA) (Megazyme, Bray, County Wicklow, Ireland).

### 4.7. Statistical Analysis

The experimental data are the mean of 3 replicates and standard error (mean ± SE). OAPnuke 1.5.2 software, HISAT 2.1.0 software, Bowtie 2 2.2.5, RSEM 2.0 software and KOBAS 2.0 software were used in data analysis. Origin 2020 and Graphpad Prism 8 were used for drawing. Pearson correlation analysis were performed on Se and associated metals in rice, and the significance level was set at a *p*-value of <0.05.

## 5. Conclusions

This study provides a comprehensive investigation into the physiological and transcriptomic effects of varying Se concentrations on rice seedlings, with a particular focus on starch and sucrose, Cys and Met metabolic pathways. The findings confirm the dual role of Se in plant growth, where a low Se concentration (1 µM) enhances shoot and root growth, promotes antioxidant activity, and upregulates genes involved in critical metabolic pathways, contributing to improved carbohydrate and amino acid synthesis. In contrast, a high Se concentration (10 µM) exerts toxic effects, inhibiting growth, suppressing metabolic activities, and downregulating key genes, highlighting the delicate balance required for Se supplementation in rice cultivation. Key metabolic pathways, including glutathione metabolism, starch and sucrose metabolism, and Cys and Met metabolism, showed significant differential gene expression under varying Se treatments. Several key genes were also identified in these metabolic processes. This study underscores the potential of low Se treatment for biofortification strategies to improve nutritional quality while minimizing toxic effects. These findings advance the understanding of Se-induced molecular mechanisms in rice and offer valuable insights for enhancing crop stress resilience and nutritional value through optimized Se management.

## Data Availability

The data of this study are available from the corresponding author, [Y.X.], upon request.

## Supplementary Materials

The following supporting information can be downloaded at: https://www.mdpi.com/article/10.3390/ijms26041596/s1.
