# Supplementary material for: Physiological and Transcriptome Analysis Provide Insights into the Effects of Low and High Selenium on Methionine and Starch Metabolism in Rice Seedlings
Source: Int J Mol Sci. 2025 Feb 13;26(4):1596. doi: 10.3390/ijms26041596 (PMC11855298; doi:10.3390/ijms26041596)
Supplement: Supplementary file 1 [file ijms-26-01596-s001.zip › ijms-3376488-supplementary.pdf]

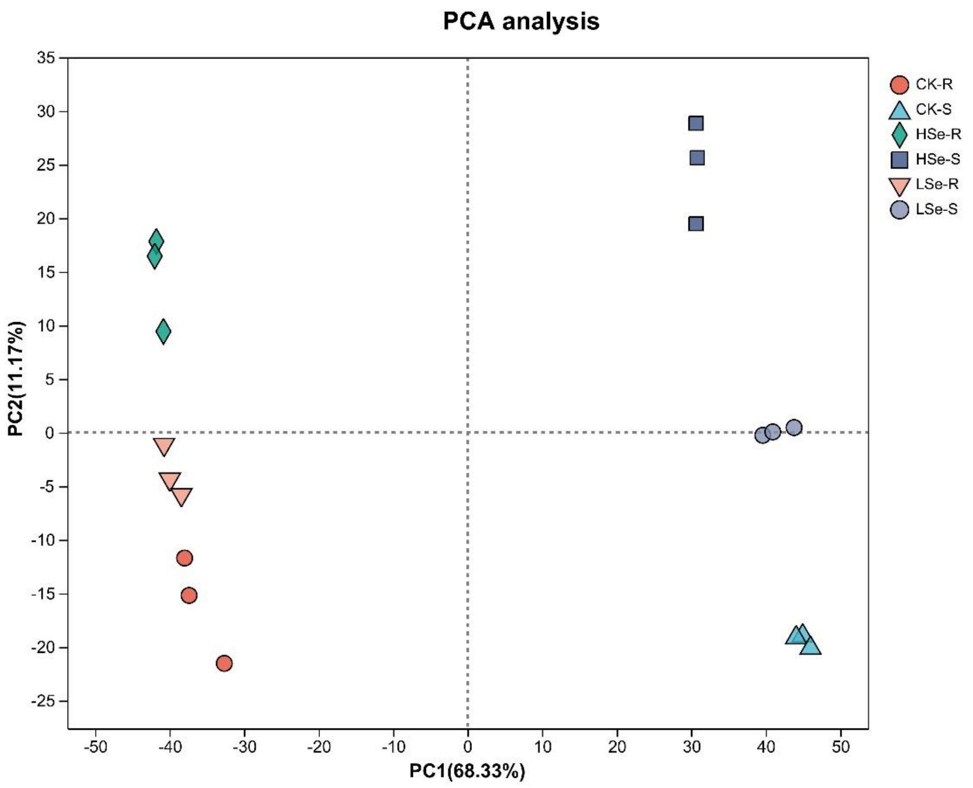

Figure S1. Principal component analysis (PCA) of gene expression in rice roots and shoots under CK, LSe, and HSe treatments. PC1 and PC2 explain 68.33% and 11.17% of the variance, respectively. Each shape and color represent a different treatment group, showing clear separation between conditions and indicating distinct gene expression patterns in response to selenium treatments.

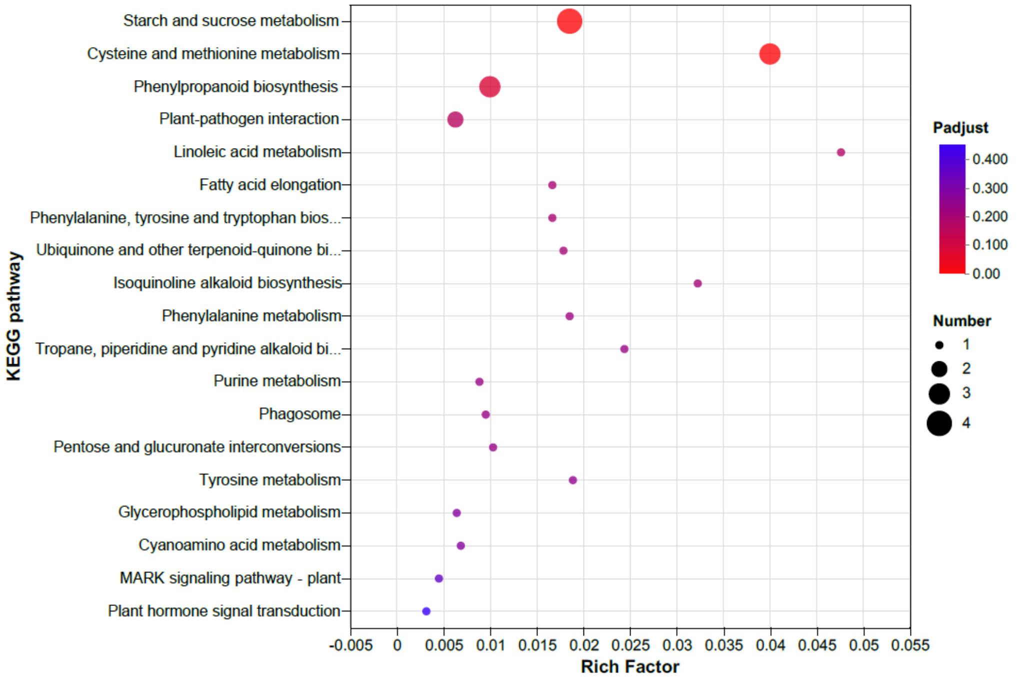

Figure S2. KEGG pathway enrichment analysis based on genes upregulated under LSe treatment and downregulated under HSe treatment compared to CK in rice roots. The x-axis represents the rich factor, indicating the proportion of differentially expressed genes (DEGs) in each pathway, while the y-axis lists the enriched KEGG pathways. Dot size reflects the number of DEGs, and color denotes the adjusted p-value (Padjust), with red indicating higher significance.

Table S1. Primer sequences of qRT-PCR

Gene	Primer sequence (5’-3’)
OsActin	F：AAGACGGTGGTTCGATGAGAGC<br>R: TGATCCAAGGCTCCTGTCCTCC
LOC_Os02g51070	F：ACCGGAACTACAAGGAGAGC<br>R：ACTGGTACTTGGCCTTGACG
LOC_Os03g11420	F：CGTCAGCCTCCCATTCAAGA<br>R：GTACACTGGTGGGCTGTTGT
LOC_Os04g39880	F：AGGCTTCCGTGACTTGCTAC<br>R：GGAAAGGAGGTGCTTGTGGT
LOC_Os04g43400	F：ACAACATCCTGCTCTCCCAC<br>R：TCAGCGGCTCGTACCATTTC
LOC_Os02g19970	F：CACACAAGCCAGAGGGATCA<br>R：ATGGCGAAAGTGATGCGAAC
LOC_Os09g25620	F：CGACCTTGGATGGCTGGAAC<br>R：ATGGCGAAAGTGATGCGAAC

Table S2. Summary of RNA-seq data quality and mapping statistics for rice samples under CK, LSe, and HSe treatments in roots and shoots.

Sample		Raw Reads	Clean Reads	Q30 (%)	GC (%)	Total Mapped	Uniquely Mapped
Root	CK	42563394	42631096	95.92	50.64	39159538 (92.85%)	37460451 (88.82%)
	LSe	43099981	42715467	95.53	52.40	41458913 (97.06%)	39258824 (91.91%
	HSe	43169424	42738902	95.56	51.54	41399270 (96.87%)	39708373 (92.91%)
Shoot	CK	42685457	42263641	95.20	53.88	41516551 (98.23%)	39269860 (92.92%)
	LSe	42873779	42478827	95.48	52.58	41732245 (98.24%)	39743816 (93.54%)
	HSe	42286647	41877433	95.32	53.06	41144745 (98.25%)	38910955 (92.91%)
